# Prevalence and socio-demographic associations of diet and physical activity risk-factors for cardiovascular disease in Bo, Sierra Leone

**DOI:** 10.1186/s12889-021-11422-3

**Published:** 2021-08-10

**Authors:** Tahir Bockarie, Maria Lisa Odland, Haja Wurie, Rashid Ansumana, Joseph Lamin, Miles Witham, Oyinlola Oyebode, Justine Davies

**Affiliations:** 1 Warwick Medical School, University of Warwick, Coventry, CV4 7AL UK; 2 Institute of Applied Health Research, College of Medical and Dental Sciences, University of Birmingham, Birmingham, B15 2TT UK; 3 College of Medicine and Allied Health Sciences, University of Sierra Leone, Freetown, Western Area Sierra Leone; 4 School of Community Health Sciences, Njala University, Bo Campus, Bo, Sierra Leone; 5 Mercy Hospital Research Laboratory, Bo, Sierra Leone; 6 AGE Research Group, NIHR Newcastle Biomedical Research Centre, Newcastle University, Newcastle upon Tyne, UK; 7 Newcastle upon Tyne Hospitals Trust, Newcastle upon Tyne, UK; 8 MRC/Wits Rural Public Health & Health Transitions Research Unit (Agincourt), University of the Witwatersrand, Johannesburg, South Africa; 9 Department for Global Health, Centre for Global Surgery, Stellenbosch University, Stellenbosch, South Africa

## Abstract

**Background:**

Little is known about modifiable dietary and physical activity risk factors for cardiovascular diseases (CVDs) in Sierra Leone. This information is critical to the development of health improvement interventions to reduce the prevalence of these diseases. This cross-sectional study investigated the prevalence and socio-demographic correlates of dietary and physical activity risk behaviours amongst adults in Bo District, Sierra Leone.

**Methods:**

Adults aged 40+ were recruited from 10 urban and 30 rural sub-districts in Bo. We examined risk factors including: ≤150 min of moderate or vigorous-intensity physical activity (MVPA) weekly, physical inactivity for ≥3 h daily, ≤5 daily portions of fruit and vegetables, and salt consumption (during cooking, at the table, and in salty snacks). We used logistic regression to investigate the relationship between these outcomes and participants’ socio-demographic characteristics.

**Results:**

1978 eligible participants (39.1% urban, 55.6% female) were included in the study. The prevalence of behavioural risk factors was 83.6% for ≤5 daily portions of fruit and vegetables; 41.4 and 91.6% for adding salt at the table or during cooking, respectively and 31.1% for eating salty snacks; 26.1% for MVPA ≤150 min weekly, and 45.6% for being physically inactive ≥3 h daily. Most MVPA was accrued at work (nearly 24 h weekly). Multivariable analysis showed that urban individuals were more likely than rural individuals to consume ≤5 daily portions of fruit and vegetables (Odds Ratio (OR) 1.09, 95% Confidence Interval (1.04–1.15)), add salt at the Table (OR 1.88 (1.82–1.94)), eat salty snacks (OR 2.00 (1.94–2.07)), and do MVPA ≤150 min weekly (OR 1.16 (1.12–1.21)). Male individuals were more likely to add salt at the Table (OR 1.23 (1.20–1.27)) or consume salty snacks (OR 1.35 (1.31–1.40)) than female individuals but were less likely to report the other behavioural risk-factors examined. Generally, people in lower wealth quintiles had lower odds of each risk factor than those in the higher wealth quintiles.

**Conclusion:**

Dietary risk factors for CVD are highly prevalent, particularly among urban residents, of Bo District, Sierra Leone. Our findings highlight that forthcoming policies in Sierra Leone need to consider modifiable risk factors for CVD in the context of urbanisation.

## Background

Cardiovascular disease (CVDs – for example, ischaemic heart disease and stroke) are projected to remain the single leading cause of death worldwide. By 2030, it is expected that almost 23.6 million people globally will die from CVDs, many from heart disease and strokes [1]. Whilst deaths from CVD have declined in the past three decades in high-income countries, partly due to action to reduce behavioural risk factors, in low-and-middle-income countries (LMICs) the rates of CVD deaths have increased over the same period [2–4]. The rise in CVD cases and deaths in sub-Saharan Africa (SSA) is attributed to rapid changes in diets and lifestyle, where traditional diets and behaviours are being replaced with diets based on processed foods and physical activity is declining with the rise in sedentary jobs and motorised transport [5–7]. In some countries in West Africa, there is evidence indicating changes to dietary patterns and physical activity, including low consumption of fruit and vegetables, increasing intake of salt, and high levels of physical inactivity [8]. Although the consequences of these risk factors are unknown for West African countries due to gaps in surveillance of CVD, it is expected that they are associated with future CVD, even if the magnitude of that association remains unknown [9]. For other countries in West Africa, there are no data even on the prevalence of risk factors; one such country is Sierra Leone [10].

Sierra Leone is a low income country on the west coast of Africa undergoing rapid economic development and urbanisation; it has 16 Districts [11]. In 2015, Sierra Leone’s national population growth rate reached 3.2% per annum while economic growth was projected to be 4.8% in 2019 [12]. The Western Area (the District which contains the capital city, Freetown) represents the bulk of the urban areas and acts as a hub for economic migration from other regions of the country. In other emerging urban areas, there has been significant intercensal change. For example, Bo District (which contains Bo city – the second largest in Sierra Leone) is located in the southern province of Sierra Leone, and between 2004 and 2015, Bo district experienced a 2.0% per annum growth rate along with the second-highest national population doubling time of 21.6 years [13].

Just as Sierra Leone’s epidemiological transition remains untracked, there is little data available on behavioural risk factors of CVD among adults in the country. Understanding these changes is of great importance for CVD prevention and control. This cross-sectional study aimed to examine the behavioural risk factors for CVD (i.e., diet and physical activity) and to determine the socio-demographic associations of these risk factors. We previously reported that the prevalence of physiological CVD risk factors such as hypertension and diabetes in Sierra Leonean adults over 40 is high at 50 and 4%, respectively [14]. It is important to develop strategies to prevent as well as manage such conditions. This study will inform potential approaches for prevention of modifiable behavioural risk-factors leading to CVD and other NCDs.

## Methods

### Study design and population

The data presented in this study are from a cross-sectional household survey conducted in Bo district, Sierra Leone between September, and December 2018. The study methods have been described previously [14]. The last population census in 2015 recorded there to be 575,478 inhabitants within the district, of which 66.1% (380,307) were in rural areas and 33.9% within urban areas, mostly in Bo city [15]. The population living in Bo is similar to other districts across Sierra Leone, with the exception of Freetown. The target population for our study were people aged 40+ years, given the likelihood that this group would have a greater prevalence of CVD than younger age groups, in-line with other previous studies [16]. The 2015 census noted that up to 17.4% (100,188) of the population were over 40 years of age in Bo district [15]. A sample size of 1893 participants was targeted to allow detection of diabetes prevalence (the CVD risk factor thought likely to have the lowest prevalence) of 4% with a precision of ±1%. We oversampled by 20% to factor for non-response and missing data.

Of the 16 chiefdoms in Bo district, most are rural, and only two, Bo City and Tikonko are urban [15]. We estimated 700 participants were required from urban areas and 1300 from rural areas in order for the sample to be representative of the urban: rural population ratio of Bo district. We purposively sampled the only two urban chiefdoms (Bo City and Tikonko). Out of the 24 eligible urban communities within Bo City and Tikonko, seven were selected from a randomly ordered list, with 100 participants recruited in each.

Out of the 14 rural chiefdoms in Bo district, seven were selected from a randomly ordered list for study. Communities (consisting of settlements or villages) within these chiefdoms were identified and two randomly selected from each Chiefdom. To attain the required sample size, in each rural community, 93 participants were recruited.

In both urban and rural areas selected for study, if the quota number of participants was not achieved in the first community, the next randomly ordered community was selected. Data collection proceeded in each community with data collectors beginning at randomly chosen points within each area and sampling from every second household from that point whilst walking along a road or track. Each household was permitted to enter no more than two people over 40 into the study. In communities with fewer than 93 people aged over 40, all people over 40 years were sampled. The geographical radius of the study was limited to 40 km from the centre of Bo to ensure accessibility for researchers. All chiefdoms and subdistricts in Bo are contained within this radius.

Data were collected electronically using ODK® (Open Data Kit) software on tablet devices by 15 trained data collectors. Questions on diet and physical activity were based on the WHO STEPwise survey [17]. For diet, participants were asked “*in a typical week, on how many days do you eat fruit or vegetables?*” and as a follow up “*how many servings of fruit and vegetables do you eat on one of those days?*”. A show card was used to enable participants to determine how many 80 g servings of fruit and vegetables they ate daily. In addition to these, participants were asked: “in the last growing seasons (May/June), how many servings of fruits and vegetables did you eat in a typical day?”. Three questions were asked to examine the salt intake. Participants were asked whether they did the following, always, often, sometimes, or never (i) add salt to food right before eating it (at the table); (ii) add salt or salty seasoning in cooking; (iii) eat salty snacks or fast food.

For physical activity, participants were asked a series of questions to find out in a typical week how many days they do moderate or vigorous physical activity as part of their (i) work, (ii) travel, (iii) leisure-time. For example, for the physical activity category, work, participants were first asked participants “does your work involve a vigorous activity that causes large increases in breathing or heart rate (like carrying, pushing, or lifting heavy loads; cycling; digging; or construction work) for at least 10 minutes at a time?” followed by another question “in a typical week, on how many days do you do vigorous-intensity activities as part of your work?” and then “how much time do you spend doing vigorous-intensity activities at work on a typical day?”. This was based on the Global Physical Activity Questionnaire [18]. Data collectors were trained to described to participants what each mode of physical activity would be using local examples when probing for response to survey questions. For sedentary activities, participants were asked: “excluding sleeping, how much time do you usually spend sitting or reclining on a typical day?”

The survey questionnaire also contained questions on demographic characteristics. Birthdate was self-reported and where participants did not know this, birth year was estimated using prompts such as key historical events. Sex was self-reported by participants. Education was reported as completion of primary, secondary, or higher education. Rural or urban area of residence was based on the sampling frame.

The survey questions were written in English, but interviews were conducted with participants in their local language of preference (either Krio or Mende).

### Measures

#### Diet

The number of proportions of fruit and vegetables eaten each day were recoded into two categories for (i) ≤ 5 portions a day and (ii) ≥5 portions a day, based on WHO guidance that people should aim to eat at least 400 g (equivalent to 5 × 80 g servings) daily [19, 20]. Salt intake (at the table, in cooking, or salty snacks) was categorised (i) Yes (always, often, sometimes) and (ii) No (included never).

### Physical activity

The total time spent by participants each week engaged in moderate physical activity was added to the twice the total time spent engaged in vigorous activity to derive the variable “total moderate and vigorous physical activity (MVPA)”. Based on WHO guidance on physical activity for health [21], the new total MVPA variable was then recoded into two categories: (i) MVPA < 150 min and (iii) MVPA ≥ 150 min per week. Physical inactivity (sedentary behaviour) was defined using a threshold of at least three hours of physical inactivity (time spent sitting or reclining when not asleep) or more per day, in line with previous publications [22].

### Covariates

Geographic area was categorised as rural or urban, sex as female or male, level of education as some education (primary school, secondary, or higher education), or no education (no formal schooling) and marital status as “married or cohabiting” (currently married or living together) or not (single, widowed, or  divorced). Age was collected as a continuous variable and grouped into five categories: 40–49, 50–59, 60–69, 70–79, 80 + .

Wealth quintiles were derived from a principal component analysis of household assets and construction materials using Filmer and Pritchett’s method [23].

### Statistical analysis

In our analysis, we excluded cases missing data on any covariate. We conducted a descriptive analysis of the socio-demographic characteristics of the study participants presented as frequency and percentage for categorical variables and mean and standard deviation for continuous variables, after assuring that distributions were normal. Probability weights for age and sex in Bo were calculated using the 2015 Population and Household Census [13]. Descriptive statistics are given using both unweighted and weighted variables to both present the characteristics of our study participants and show the representative prevalence for the Bo district population.

Multivariable logistic regression models were fitted using forced entry with covariables selected based on their known relationship with the dependent variables [24–28]. *P*-values less than 0.05 were considered to indicate statistical significance. All analyses were undertaken on SPSS version 26 (Statistical Package for the Social Sciences). All methods were performed in accordance with the relevant guidelines and regulations.

## Results

Given the difficulty in communication between data collectors working in remote rural areas, we exceeded the target sample size for this study and the number of participants with data totalled 2071. From this total, 93 adults were excluded from analyses due to missing values in the socio-demographic co-variables, leaving 1978 individuals (55.6% females and 44.4% males, unweighted) with complete data on sociodemographic factors. Demographic characteristics of the study participants are shown, and weighted percentages reported in Table 1. Our weighted sample comprises 62.6% living in rural areas, with 37.4% in urban areas; 72.6% were either married or cohabiting; 67.3% were categorised as being uneducated, and 32.7% reported as having some education. The  mean age was 57 years (SD 13.52); and a large proportion (44.5%) of the population were within the age group 40–49.

**Table 1:** Socio-demographic characteristics of study population

Household Survey Participants		
	Unweighted	Weighted
	n (%)	n (%)
**Location**		
Rural	1205 (60.9)	60,739 (62.6)
Urban	773 (39.1)	36,217 (37.4)
**Sex**		
Female	1100 (55.6)	47,766 (49.3)
Male	878 (44.4)	49,190 (50.7)
**Age Group**		
40–49	682 (34.5)	43,116 (44.5)
50–59	546 (27.6)	24,379 (25.1)
60–69	340 (17.2)	14,381 (14.8)
70–79	251 (12.7)	8913 (9.2)
80+	159 (8.0)	6166 (6.4)
**Education**		
Some Education	615 (31.1)	31,731 (32.7)
No Education	1363 (68.9)	65,225 (67.3)
**Marital Status**		
Married or Cohabiting	1355 (68.5)	70,364 (72.6)
Not Married	623 (31.5)	26,591 (27.4)
**Wealth Quintile**		
Poorest	395 (20.0)	19,896 (20.5)
Poorer	397 (20.1)	19,964 (20.6)
Middle	397 (20.1)	19,493 (20.1)
Richer	394 (19.9)	19,093 (19.7)
Richest	395 (20.0)	18,510 (19.1)

Use of salt in cooking was widespread, as shown in Table 2. Weighted data shows more than 9 out of 10 (91.9%) of this population added some type of salt, or seasoning that contains salt (sometimes, often, or always) when cooking. Salt was added at the table before eating by 40.9%, while 30.9% ate salty snacks. Of the population, 83.2% ate fewer than five portions of fruit or vegetables per day*.* The mean portions of fruit and vegetables consumed was 3.3 (SD = 1.66). MVPA of ≥ 150 min per week was achieved by 77.4% of this population, and 44.0% were physically inactive for ≥ 3 h per day. On average, 40 h, and 44 min (SD = 46 h, 40 min) of MVPA were accrued per week (Table 3). Fewer hours of physical activity were for leisure, whereas a far greater proportion of physical activity was spent at work or for transport. On average, for those who did occupational physical activity, 23 h and 47 min of physical activity per week were accrued (SD = 26 h, 19 min). Participants who achieved physical activity through travel and during leisure time, spent 6 h and 10 min per week engaged in walking and cycling for travel  (SD = 9 h, 0 min) and 31 min per week on leisure activities (SD = 3 h, 33 min) on average.

**Table 2:** Behavioural risk factors summary descriptive

	Unweighted	Weighted		
**Salt Cooking**	n (%)	n (%)	Females n (%)	Males n (%)
Yes	1810 (91.6)	89,077 (91.9)	43,448 (91.0)	45,629 (92.8)
No	167 (8.4)	7845 (8.1)	4284 (9.0)	3560 (7.2)
**Salt Table**	n (%)	n (%)	Females n (%)	Males n (%)
Yes	818 (41.4)	39,638 (40.9)	18,648 (39.2)	20,990 (42.9)
No	1150 (58.4)	56,904 (58.7)	28,968 (60.8)	27,936 (57.1)
**Salty Snacks**	n (%)	n (%)	Females n (%)	Males n (%)
Yes	590 (31.1)	28,766 (30.9)	13,017 (28.4)	15,749 (33.3)
No	1307 (68.9)	64,405 (69.1)	32,872 (72.6)	31,532 (66.7)
**Fruit and Vegetables**	n (%)	n (%)	Females n (%)	Males n (%)
<5FVeg	1131 (83.6)	55,307 (83.2)	27,814 (86.3)	27,492 (80.2)
≥5FVeg	222 (16.4)	11,171 (16.8)	4398 (13.7)	6773 (19.8)
**Physical Activity MVPA**	n (%)	n (%)	Females n (%)	Males n (%)
<150mins	516 (26.1)	21,927 (22.6)	14,243 (29.8)	7684 (15.6)
≥150mins	1462 (73.9)	75,028 (77.4)	33,523 (70.2)	41,505 (84.4)
**Physical Inactivity**	n (%)	n (%)	Females n (%)	Males n (%)
≥3 h	902 (45.6)	42,655 (44.0)	22,486 (47.1)	20,168 (41.0)
<3 h	1076 (54.4)	54,301 (56.0)	25,280 (52.9)	29,021 (59.0)
**Combined Risk Factors**	n (%)	n (%)	Females n (%)	Males n (%)
≤1 Risk Factors	169 (8.5)	9036 (9.3)	3284 (6.9)	5752 (11.7)
2 Risk Factors	571 (28.9)	29,098 (30.0)	14,555 (30.5)	14,542 (29.6)
3 Risk Factors	663 (33.5)	32,290 (33.3)	16,489 (34.5)	15,801 (32.1)
4 Risk Factors	431 (21.8)	20,257 (20.9)	10,012 (21.0)	10,244 (20.8)
5 Risk Factors	117 (5.9)	5198 (5.4)	2712 (5.7)	2486 (5.1)
6 Risk Factors	27 (1.4)	1077 (1.1)	713 (1.5)	365 (0.7)

**Table 3:** Physical Activity risk factor summary descriptive

Physical activity	Mean (hrs: mins)	Std. Deviation
**Category: Work**		
Vigorous PA work per week	17:21	20:32
Moderate PA work per week	6:53	13:30
**Category: Travel**		
Moderate PA per week	06:05	08:22
Vigorous PA per week	00:04	01:52
**Category: Leisure**		
Leisure- Vigorous PA per week	00:17	02:43
Leisure- Moderate PA per week	00:14	01:44
**Total MVPA**		
Work PA per week	23:47	26.19
Travel PA per week	06:10	09:00
Leisure PA per week	00:31	03:33
**Category: Total Activity**		
Total Vigorous PA per week	14:34	20:06
Total Moderate PA per week	11:35	15:10
Sedentary hours per day	03:08	02:34
MVPA in hours per week	40:44	46:40

In our sample, almost all participants had at least one modifiable risk factors (99.8%) with the majority (62.6%) experiencing 3 or more of the 6 modifiable risk factors (< 5 portions of fruit and vegetables daily; salt at table; salt in cooking; eating salty snacks; < 150 MVPA; ≥3hous physical inactivity) investigated (Table 2).

**Fig. 1:**
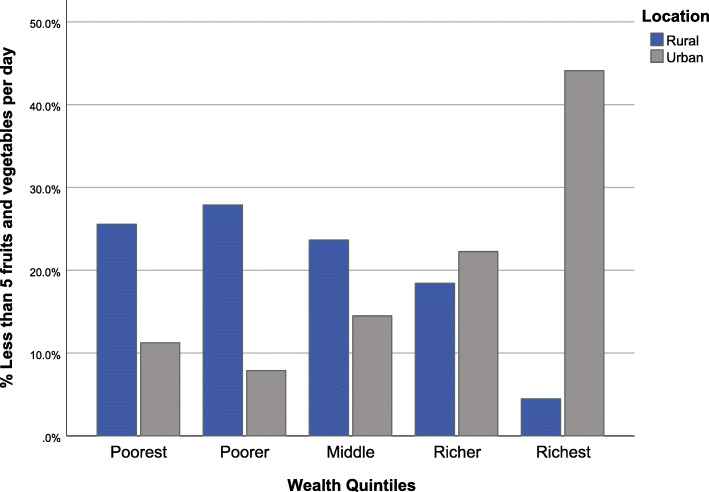
Less than five fruit and vegetables per day by location and wealth

Multivariable logistic regression shows that urban dwellers (OR = 1.09, 95%CI (1.04–1.15), *p * < 0.001) had higher odds of insufficient fruit and vegetable consumption. Compared with females, males had lower odds (OR = 0.78, 95%CI (0.74–0.82), *p* < 0.001) of insufficient fruit and vegetable consumption, whilst individuals with no education compared with those with some education had higher odds (OR = 1.41, 95%CI (1.35–1.48), *p * < 0.001). Compared to married individuals, those who were unmarried had higher odds of insufficient fruit and vegetable consumption. Odds of insufficient consumption increased with wealth, and there was no clear association with age (Table 4). Figure 1 shows the percentage of people with insufficient fruit and vegetable consumption by rural or urban location and wealth.

**Table 4:** Association between demographic characteristics and diet and exercise

	< 5Fruit and vegetables per day		Salt table		Salt cooking		Salty snacks		MVPA< 150MIN per week		Physical inactivity ≥ 3 h per day	
	OR (95% CI)	***p-value***	OR (95% CI)	***p-value***	OR (95% CI)	***p-value***	OR (95% CI)	***p-value***	OR (95% CI)	***p-value***	OR (95% CI)	***p-value***
**Location**												
Rural	Referent	–	Referent	–	Referent	–	Referent	–	Referent	–	Referent	–
Urban	1.09 (1.04–1.15)	0.001	1.88 (1.82–1.94)	< 0.001	0.41 (0.39–0.43)	< 0.001	2.00 (1.94–2.07)	< 0.001	1.16 (1.12–1.21)	< 0.001	0.94 (0.91–0.97)	< 0.001
**Sex**												
Female	Referent		Referent		Referent		Referent		Referent		Referent	
Male	0.78 (0.74–0.82)	< 0.001	1.23 (1.20–1.27)	< 0.001	0.89 (0.84–0.94)	< 0.001	1.35 (1.31–1.40)	< 0.001	0.63 (0.61–0.66)	< 0.001	0.90 (0.87–0.93)	< 0.001
**Age Group**												
40–49	Referent	–	Referent	–	Referent	–	Referent	–	Referent	–	Referent	–
50–59	1.06 (1.01–1.12)	0.029	1.21 (1.17–1.25)	< 0.001	0.81 (0.77–0.86)	< 0.001	1.24 (1.20–1.29)	< 0.001	1.94 (1.85–2.02)	< 0.001	1.06 (1.03–1.10)	< 0.001
60–69	0.98 (0.92–1.04)	0.483	1.35 (1.30–1.40)	< 0.001	0.83 (0.78–0.89)	< 0.001	1.49 (1.43–1.56)	< 0.001	2.91 (2.77–3.06)	< 0.001	1.32 (1.27–1.37)	< 0.001
70–79	1.30 (1.20–1.41)	< 0.001	1.44 (1.37–1.51)	< 0.001	0.85 (0.79–0.93)	< 0.001	1.32 (1.25–1.40)	< 0.001	6.20 (5.87–6.55)	< 0.001	1.93 (1.84–2.02)	< 0.001
80+	0.44 (0.41–0.47)	< 0.001	1.18 (1.11–1.24)	< 0.001	2.00 (1.76–2.27)	< 0.001	0.67 (0.62–0.72)	< 0.001	9.64 (9.05–10.27)	< 0.001	3.24 (3.05–3.44)	< 0.001
**Education**												
Some education	Referent		Referent		Referent		Referent		Referent		Referent	
No education	1.41 (1.35–1.48)	< 0.001	0.94 (0.91–0.97)	< 0.001	0.87 (0.82–0.91)	< 0.001	0.69 (0.66–0.71)	< 0.001	1.14 (1.10–1.19)	< 0.001	1.13 (1.10–1.16)	< 0.001
**Marital Status**												
Married	Referent	–	Referent	–	Referent	–	Referent	–	Referent	–	Referent	–
Not Married	1.38 (1.30–1.46)	< 0.001	1.00 (0.96–1.03)	0.807	0.65 (0.61–0.68)	< 0.001	0.87 (0.84–0.90)	< 0.001	1.83 (1.77–1.91)	< 0.001	1.21 (1.17–1.25)	< 0.001
**Wealth Quintile**												
Poorest	Referent	–	Referent	–	Referent	–	Referent	–	Referent	–	Referent	–
Poorer	1.14 (1.07–1.22)	< 0.001	1.03 (0.99–1.07)	0.159	1.50 (1.38–1.62)	< 0.001	1.07 (1.01–1.13)	< 0.001	0.97 (0.92–1.03)	0.367	1.84 (1.77–1.92)	< 0.001
Middle	1.17 (1.09–1.25)	< 0.001	1.18 (1.13–1.22)	< 0.001	1.41 (1.30–1.53)	< 0.001	1.95 (1.86–2.05)	< 0.001	1.51 (1.43–1.60)	< 0.001	1.33 (1.28–1.39)	< 0.001
Richer	1.29 (1.20–1.38)	< 0.001	1.11 (1.06–1.16)	< 0.001	0.96 (0.89–1.04)	0.322	2.29 (2.17–2.40)	< 0.001	1.42 (1.34–1.50)	< 0.001	1.31 (1.25–1.36)	< 0.001
Richest	1.29 (1.19–1.39)	< 0.001	0.58 (0.56–0.61)	< 0.001	1.05 (0.97–1.14)	0.357	2.24 (2.12–2.37)	< 0.001	2.13 (2.00–2.26)	< 0.001	1.81 (1.73–1.90)	< 0.001

Salt intake at the table, in cooking, and salty snacks varied across demographic and socio-economic characteristics (Table 4). In the case of salt at the table, urban dwellers (OR = 1.88, 95%CI (1.82–1.94), *p * < 0.001) and males (OR = 1.23, 95%CI (1.20–1.27), *p * < 0.001) had higher odds of this risk-factor compared with each reference category. Individuals with no education reported lower odds compared to individuals with some education (OR = 0.94, 95%CI (0.91–0.97), *p * < 0.001). The adding of salt at the table generally increased with age. The middle three wealth categories had high odds of adding salt at the table compared with the poorest quintile, but the richest quintile had the lowest odds of all (5th wealth quintile, OR = 0.58, 95%CI (0.56–0.61), *p * < 0.001) (Table 4).

For salt in cooking, urban individuals had lower odds (OR = 0.41, 95%CI (0.39–0.43) *p * < 0.001) compared to rural individuals, while male individuals had lower odds compared to female individuals (OR = 0.89, 95%CI (0.84–0.94), *p * < 0.001). Uneducated individuals had lower odds of adding salt in cooking (OR = 0.87, 95%CI (0.82–0.91), *p* < 0.001). Unmarried individuals (compared with married) reported lower odds of adding salt in cooking (OR = 0.65, 95%CI (0.61–0.68), *p * < 0.001) (Table 4).

The odds of consuming salty snacks were higher in urban individuals than rural individuals (OR = 2.00, 95%CI (1.94–2.07), *p * < 0.001) and male individuals compared with female individuals (OR = 1.35, 95%CI (1.31–1.40), *p * < 0.001). Having no education (OR = 0.69, 95%CI (0.66–0.71), *p* < 0.001) and not being married (OR = 0.87, 95%CI (0.84–0.90), *p =* 0.001) was associated with lower odds of eating salty snacks compared with relevant reference categories. Consumption of salty snacks generally increased with age and wealth, richer households consumed the most (4th Wealth Quintile, OR = 2.29, 95%CI (2.17–2.40), *p * < 0.001) (Table 4).

**Fig. 2:**
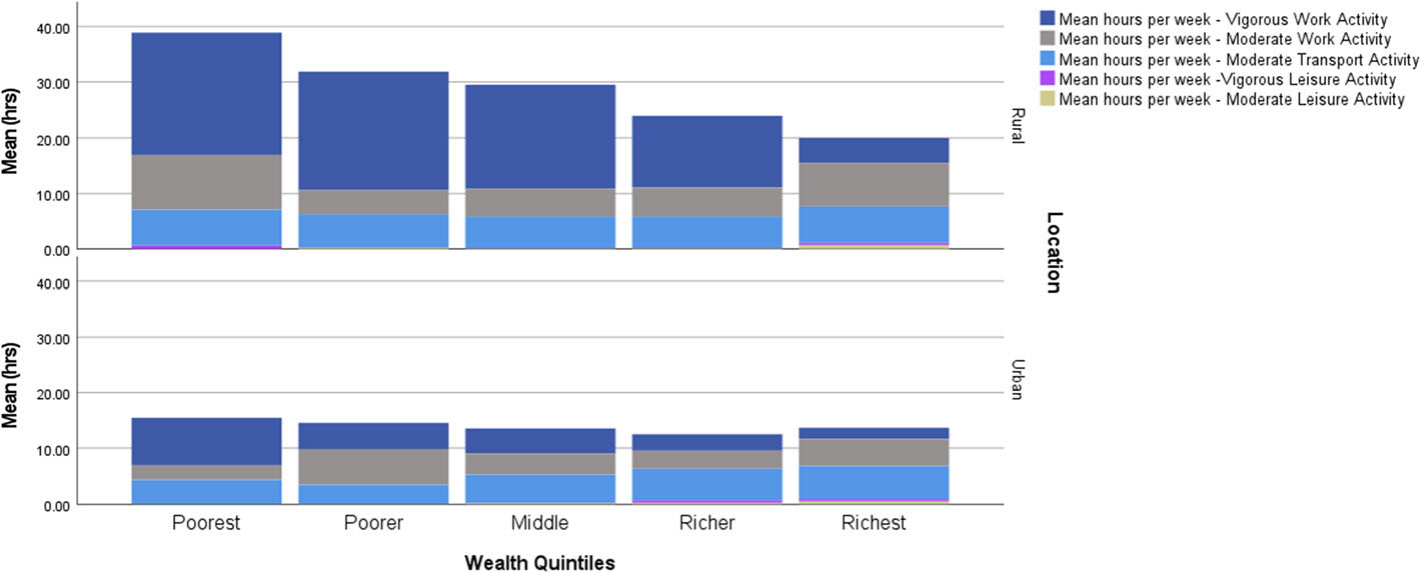
Average number of hours per week of different types of vigorous or moderate physical activity by location and wealth quintiles

In the multivariable analysis, living in an urban area was associated with higher odds of doing < 150 min of MVPA per week compared with living in a rural area (OR = 1.16, 95%CI (1.12–1.21) *p* < 0.001). Individuals with no education had higher odds of doing < 150 min of MVPA per week (OR = 1.14, 95%CI (1.10–1.19), *p * < 0.001) compared to individuals with some education. Being unmarried was associated with higher odds of doing < 150 min of MVPA per week (OR = 1.84, 95%CI (1.77–1.91), *p * < 0.001). Increasing age was associated with higher odds of doing < 150 min of MVPA per week, as expected the oldest individuals (80+) were least likely to accrue  ≥150 min of MVPA (OR = 9.64, 95%CI (9.05–10.27), *p * < 0.001) per week (Table 4). Figure 2 shows mean number of hours of type of MVPA by location and wealth.

**Fig. 3:**
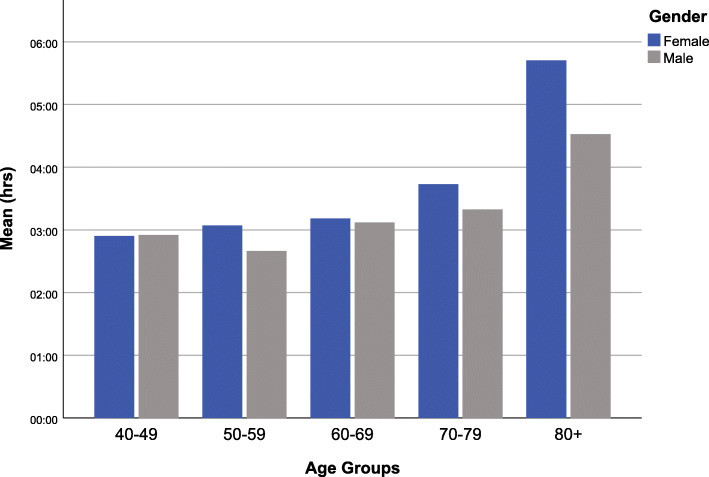
Average sedentary time (in hours) per day by age groups and sex

Multivariable analysis shows that urban individuals had lower odds (OR = 0.94, 95%CI (0.91–0.97), *p * < 0.001) of being physically inactive for ≥ 3 h per day. Similarly, male individuals had lower odds of being physically inactive for  ≥ 3 h per day (OR = 0.90, 95%CI (0.87–0.93), *p * < 0.001). Having no education was associated with higher odds of being sedentary for  ≥ 3 h per day (OR = 1.13, 95%CI (1.10–1.16), *p* < 0.001). Unmarried individuals had higher odds of being physically inactive for ≥ 3 h per day (OR = 1.21, 95%CI (1.17–1.25, *p* < 0.001). Increasing age was associated with higher odds of physical inactivity ≥ 3 h with the oldest age-groups (80+) being the most sedentary (OR = 3.24, 95%CI (3.05–3.44), *p * < 0.001). Affluence was associated with increased odds of physical inactivity across all wealth groups (Table 4). Figure 3 shows mean number of hours of sedentary time by age and sex.

## Discussion

Our study reveals that insufficient daily intake of fruit and vegetables and adding salt in cooking were highly prevalent in this population across urban and rural Sierra Leone. However, much of the population were physically active exceeding the WHO recommendations for number of minutes of physical activity per week, except for the oldest age-groups (70–79, 80+) who were more likely to have lower levels of physical activity.

Fruit and vegetable consumption have clear health and wellbeing benefits [29]. Our study used the five-a-day target because it is widely recommended that eating at least five 80 g portions of fruit and vegetables a day (400 g in total) lowers rates of cardiovascular mortality [30–32]. Other studies on fruit and vegetable consumption in LMICs have also reported less than the recommended consumption across age groups and wealth quintiles [33, 34]. A systematic review showed that daily per capita fruit and vegetable intake in populations in SSA was, on average 268 g (or 3.4 portions) [35] similar to average daily per capita intake in our study which was 3.0 portions.

We found that individuals living in urban areas were less likely than those in rural areas to meet their five-a-day for sufficient intake of fruit and vegetables [36, 37]. This urban-rural divide is in line with a systematic review done in 47 countries in SSA [35] and other studies from Mozambique [38] and Tanzania [39]. This might be due to greater access to fruit and vegetables in rural Sierra Leone, as many rural inhabitants are involved in subsistence farming [40]. However, even those in rural areas are not eating enough. This calls into question what other barriers might exist to adequate consumption. Inefficient supply chains [41] might, in part, lead to the increased cost of transporting produce to urban areas, contributing to the difference observed in our study. Another plausible reason for lower fruit and vegetable consumption in urban districts, which may also explain the association of reduced fruit and vegetable intake with increasing wealth, might be linked to the waning popularity of the traditional Sierra Leone diet. The cultural association of affluence and urbanisation with a more ‘Western’ meat-heavy diet, could explain why our study found lower intakes of fruit and vegetables among urban dwellers, particularly those living in richer and the richest households.

We noted that males were more likely than females to consume the recommended number of fruit and vegetables per day. Very little has been written about nationwide diet patterns and cultures in Sierra Leone [42]. Still, potential explanations for our findings include psychosocial factors [43], food preferences between genders [44], or cultural beliefs relating to certain foods in Sierra Leone that one gender might avoid over the other [45]. Our study is in line with literature suggesting that marriage and education are associated with healthy dietary behaviour [46–51].

Our study showed that most participants added salt to cooking, with a lower but sizeable proportion adding salt at the table or eating salty snacks. This finding agrees with a systematic review which found that average salt intake in SSA populations was above WHO recommendations [52]. The present study findings are also congruent with those of our previously published analysis of data from the same population showing a high prevalence of hypertension (51%), with prevalence in the urban population slightly higher than the rural population. Urban populations, men, older age -groups, and richer quintiles were generally more likely to report adding salt or eating salty snacks; meanwhile uneducated individuals were generally less likely to report adding salt to food, although these patterns were not always consistent across all the salt variables, and without quantitative data on salt intake, it is difficult to know for sure which populations groups are at greatest risk from excess salt consumption.

In the current study, insufficient physical activity and excessive sedentary behaviour can be viewed as two separate ways of looking at similar unhealthy behaviours. Living in an urban area was associated with higher odds of MVPA < 150 min per week,  in line with previous findings that reported a higher prevalence of insufficient physical activity for urban settings in Kenya and Cameroon [53–56]. However, in our analysis urban dwellers were less likely to be physically inactive (≥ 3 h daily). Although reduced physical activity and increased sedentary time are known to be risk factors for CVD [57–59], the interaction between the two is likely to be complex and further exploration is needed to determine the balance of risk for these factors in this population in advance of any national strategy development [53].

Time spent doing different types of activity (vigorous and moderate activity at work, vigorous and moderate activity by leisure, or activity from transport) has been the subject of previous studies  in LMICs, which have found that more time was typically accrued at work and for transport than during leisure [60–62]. The present study also showed that Sierra Leoneans spent little time conducting physical activity in leisure time, whereas a far greater proportion of physical activity was spent at work and for transport. This leaves Sierra Leoneans vulnerable to CVD risk factors if work and travel practices change. In a study of physical activity in 22 African countries [63], countries with dominant or developed transportation networks reported the lowest levels of activity by transport. Though transport activity varies from place to place in Sierra Leone, a broad policy to be implemented might discourage take-up of motorised transportation, while at the same time promoting other modes (walking and cycling) and increasing facilities for recreational leisure activities.

Our study confirms previous findings that there are lower rates of physical activity in women than men [64–66]. Understanding expectations, cultural norms and traditional economic roles played by genders in Sierra Leone could be vital to addressing sex differences in physical activity to support achieving the 2025 global physical activity target [64].

Increasing age was independently associated with higher odds of insufficient physical activity and increased sedentary lifestyle. These findings from our study are reflective of current data and trends among adults across 22 SSA countries [63, 67]. However, although the decline in physical activity with age is reflective of that seen in other populations, the levels of physical activity in older people in our sample are still higher than those seen in other populations in Africa [68]. Any proposed interventions relating to physical activity should consider age-adapted leisure activities to address physical inactivity across all age groups (more especially in older ages), and in those who are unemployed, retired or whose occupations do not involve physical activity.

In Sierra Leone, our results show the poorer and richest households reported higher odds of sedentary behaviour. In contrast, there was a clearer relationship between wealth and exercise: the richest households were the least likely to be physically active for at least 150 min per week, and poorest households were the most. There should be some concern as to whether declining rates of exercise by higher wealth quintiles is a sign of what is to come as Sierra Leone becomes wealthier and employment changes to become less physically demanding, given that physical activity was largely undertaken through work and travel which are vulnerable to mechanisation and/or office jobs.

Although our analysis has shown differences in lifestyle behaviours between different socio-economic and demographic groups, the reasons behind these differences in Sierra Leone are still at this point fairly speculative. Further research needs to be done to ascertain why these differences exist in this context and how to change behaviour. The high burden of CVD in Sierra Leone [14] shows that behaviour change is desperately needed, implementation of effective policies will need to be tailored to the local context to increase physical activity and promote the health benefits of five-a-day and salt reduction to specific groups to improve their wellbeing and to contribute towards Sierra Leone achieving the 2030 Sustainable Development Goals [69].

### Limitations

Due to the self-reporting approach used for this study, there are limitations. In terms of diet, we were unable to assess dietary energy intake or salt intake. Similarly, our approach might not offer a precise indication of intake of fruit and vegetables per day [70, 71]. One of the main sources of error in dietary assessment is typically misreporting, which can encompass under and over-reporting [70, 71]. In addition to this, consideration should be given to whether misreporting by individuals may have occurred differentially across demographic indicators such as gender. Taking this example further, if women traditionally cook in Sierra Leonean households whilst men do not [40], reporting of salt used in cooking by men may be less accurate due to a lack of first-hand knowledge.

Additionally, the survey questionnaire was written in English, and data collectors were native speakers who translated the survey into the local languages Krio and Mende. This might have led to differing explanations or interpretations of the questions between languages, especially for questions relating to physical activity by work, travel, and leisure.

Further, the salt measures included four options (always, often, sometimes, never) which were qualitative and not quantitative in measure, and the amount of salt added was not assessed so that salt behaviour rather than salt intake is reported here. Future studies quantifying sodium intake are needed. Other biases associated with reliance on self-reporting includes the time estimations provided by participants for exercise and sedentary behaviour. It is important to note that 69% of study participants had no education and conceptualising time in relation to intensity or mode of physical activity might have generated some overestimated or underestimated timings. Prospective studies looking into physical activity might adopt other approaches for accurately measuring physical activity in relation to time, such as using a visual clock to aid participants in estimating time more accurately. Lastly our study may have been subject to selection bias as females were more likely compared to males to be home during the time of the survey. To mitigate against this, our data collectors started collecting data in the early morning hours and late evenings to capture a representative proportion of males in our study.

### Strengths

Despite the limitations expressed above, this manuscript provides one of the first detailed studies into diet and physical activity risk factors for cardiovascular disease, as well as the associated socio-economic characteristics of adults in urban and rural Sierra Leone.

## Conclusion

Sierra Leone is experiencing rapid urbanisation which may drive CVD risk factors associated with diet and physical activity. This study highlights that 92% of the population add salt in cooking, and 83% consume an insufficient number of portions of fruit and vegetables per day. However, the population of Bo is by and large physically active (77% achieve ≥ 150 min of MVPA), but leisure-time physical activities must be adopted to future-proof such behaviours against changes to work, transport, or leisure cultures. To support progress towards the prevention of CVD, improvements against these risk factors in middle-later ages (broadly designed as ages 40–79) could lead to a compression of morbidity during older ages and healthier longevity. This in turn can improve quality of life as well as improving productivity and future economic growth of Sierra Leone to achieve its 2030 Agenda for Sustainable Development, in particular Goal 3, which includes as a target a one-third reduction of premature mortality from NCDs through prevention and treatment.

## Data Availability

*The datasets used and analysed during the current study available from the corresponding author on* ***reasonable request****. All enquiries are to be addressed to Professor Justine I. Davies* via *e-mail:* j.davies.6@bham.ac.uk.
