# Correction to: Prevalence and socio-demographic associations of diet and physical activity risk-factors for cardiovascular disease in Bo, Sierra Leone

**DOI:** 10.1186/s12889-021-11759-9

**Published:** 2021-10-07

**Authors:** Tahir Bockarie, Maria Lisa Odland, Haja Wurie, Rashid Ansumana, Joseph Lamin, Miles Witham, Oyinlola Oyebode, Justine Davies

**Affiliations:** 1 Warwick Medical School, University of Warwick, Coventry, CV4 7AL UK; 2 Institute of Applied Health Research, College of Medical and Dental Sciences, University of Birmingham, B15 2TT, Birmingham, UK; 3 College of Medicine and Allied Health Sciences, University of Sierra Leone, Freetown, Western Area Sierra Leone; 4 School of Community Health Sciences, Njala University, Bo Campus, Bo, Sierra Leone; 5 Mercy Hospital Research Laboratory, Bo, Sierra Leone; 6 AGE Research Group, NIHR Newcastle Biomedical Research Centre, Newcastle University, Newcastle upon Tyne, UK; 7 Newcastle upon Tyne Hospitals Trust, Newcastle upon Tyne, UK; 8 MRC/Wits Rural Public Health & Health Transitions Research Unit (Agincourt), University of the Witwatersrand, Johannesburg, South Africa; 9 Department for Global Health, Centre for Global Surgery, Stellenbosch University, Stellenbosch, South Africa

**Correction to: BMC Public Health 21, 1530 (2021)**

**https://doi.org/** **10.1186/s12889-021-11,422-3**

In Fig. 2 of this article [1] the figure panel should have appeared as shown below. The original article has been updated.

**Fig. 2:**
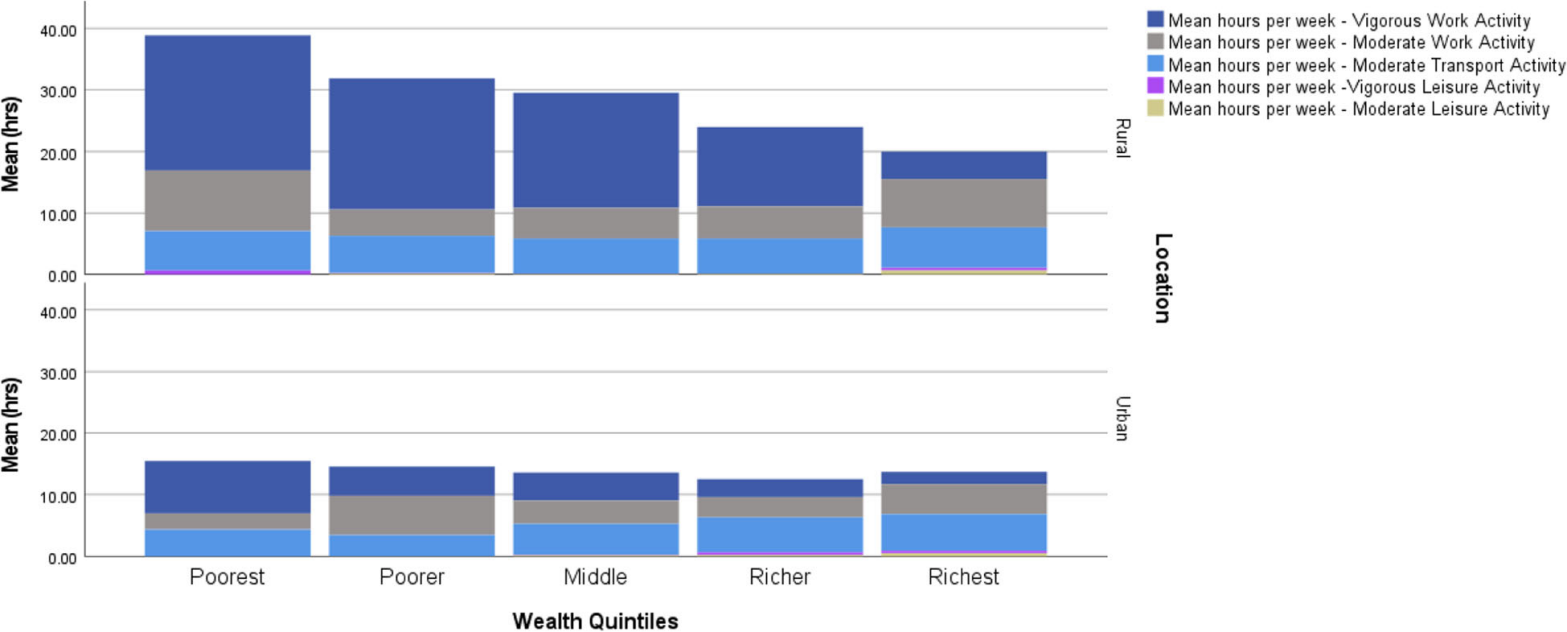
Average number of hours per week of different types of vigorous or moderate physical activity by location and wealth quintiles
